# An Account of the Practice of One of the Physicians of the Westminster General Dispensary, and of the Western Dispensary, from the 20th of September, to the 20th of October

**Published:** 1806-11

**Authors:** S. Fothergill

**Affiliations:** Southampton Street, Strand


*An Account of the Practice of one of the Physicians of the Westminster General Dispensary, and of the Western Dispensary, from the 20th of September, to the 20th of October.*

| ACUTE DISEASES. | |
|---|---|
| Erysipelas | 1 |
| Urticaria | 1 |
| Catarrh | 3 |
| Acute Rheumatism | 7 |
| Pleurisy | 1 |
| Peripneumony | 4 |
| Synochus | 5 |
| Measles | 3 |
| Chicken-Pox | 1 |
| Hooping-Cough | 1 |
| Cholera | 2 |

| | | | |
|---|---|---|---|
| Acute Diseases of Infants | 8 | Dropsy - - - | 2 |
| CHRONIC DISEASES. | | Gravel and Dysuria | 3 |
| Cough and Dyspnœa | 26 | Worms - - - - | 1 |
| Pleurodyne - - | 3 | Diarrhœa - - - | 7 |
| Pulmonary Consumption | 3 | Dysphagia - - | 1 |
| Hæmoptoe & Epistaxis | 3 | Ulcerated Throat - | 2 |
| Asthenia - - | 12 | Hernia - - - - | 2 |
| Chronic Rheumatism | 8 | Syphilis - - - - | 2 |
| Lumbago - - | 2 | Psora - - - - | 2 |
| Cephalalgia and Vertigo | 7 | Lepra - - - - | 1 |
| Paralysis - - - | 3 | Erythema - - | 1 |
| Gastrodynia - - | 6 | Amenorrhœa - - | 5 |
| Enterodynia - - | 4 | Chlorosis - - - | 3 |
| Pyrosis - - - | 2 | Menorrhagia - - | 2 |
| Constipation & Vomiting | 6 | Leucorrhœa - - | 7 |
| Dyspepsia - - | 7 | Uterine Hæmorrhage | 1 |
| Jaundice - - - | 4 | Cancer Uteri - - | 1 |

The diseases which have occurred during the last month, differed little from those of the preceding Report. The season indeed is further advanced, yet the weather continuing to be fine, the complaints which I have had an opportunity of witnessing, with two or three exceptions, have been generally mild in their symptoms, and favourable in their termination.

One case occasioned me considerable anxiety. The patient was a young woman, who was the subject of acute rheumatism and pleurisy, combined with a degree of peripneumony. When I saw her she had been ill a month, and was in a very weak state, complaining of acute pains in the chest and sides, which obliged her to lie constantly on her back; the swelling of the joints had nearly subsided, though they were still very painful; the pulse was 130; the breathing particularly frequent; the tongue was parched, brown in the middle, with white edges; and the urine was scanty, and of a reddish colour. A clammy sweat partially bedewed the skin; the cheeks were occasionally flushed, and she enjoyed very short intervals of sleep, frequently starting, her slumbers being interrupted by frightful dreams. The case had hitherto been treated as a case of rheumatism; and though the respiration was most frequent and painful, bark had been given in considerable quantity. At this period of the disease, general bleeding was inadmissible; leeches applied to the chest, and blisters, with frequent doses of antimonials, camphor, ether, and opium, gave ease, and she got some quiet sleep.

From

From this time she gradually recovered, and is now convalescent. As her pulse continued for several days at 120, digitalis was given, with a view to diminish its frequency; it did not, however, produce the desired effect, tho' taken as freely as was deemed prudent; the bowels were occasionally opened with calomel and rhubarb.

These complicated cases demand all our attention and skill; they are difficult to arrange under any order of Nosology; and he who attempts to learn from the rules and definitions of the ablest Nosologists, all the varying appearances of diseases, will understand them as little as he will know how to combat their dangerous effects. In pure peripneumony and pleuritic inflammation, copious bleeding from the arm is often speedily effectual, and, in general, is so forcibly indicated, that there can be little hesitation in adopting this potent remedy. This, however, is sometimes neglected in the first stage of the disease, from timidity on the part of the practitioner, or from want of timely application of the patient; and, in some instances, the fever assumes a typhoid character. In such cases, particularly in large populous towns, where the air is confined and impure, when the propriety of bleeding admits of any doubt, I have ever found it safest not to bleed, and have with pleasure witnessed the recovery of many patients under circumstances where, in other hands, the lancet would probably have been used.

The case of pleurisy, in the present Report, occurred in a little girl, aged two years, who died soon after I saw her. Previous to her attack, she had taken a large quantity of worm medicines from a quack, and her constitution was much enfeebled, without any worms having been discovered. Upon inspecting the body after death, we observed general adhesion of the lungs to the pleura costalis, which was a little thickened, and coagulable lymph was effused on the surface; the lungs and other viscera of the thorax were perfectly sound; and in the abdomen, the only morbid appearance was the pancreas, considerably enlarged, and nearly scirrhous. Thus, in their eagerness to cure their child of a complaint which did not exist, the parents neglected the real and unequivocal symptoms of danger; confiding in the efficacy of empirical nostrums, whilst the last pulse of life was yielding to an acute disease.

S. FOTHERGILL.

*Southampton Street, Strand, Oct.* 21, 1806.

